# Natural Melanogenesis Inhibitors Acting Through the Down-Regulation of Tyrosinase Activity

**DOI:** 10.3390/ma5091661

**Published:** 2012-09-17

**Authors:** Te-Sheng Chang

**Affiliations:** Department of Biological Sciences and Technology, National University of Tainan, 33, Sec. 2, Shu-Lin St., Tainan 700, Taiwan, China; E-Mail: mozyme2001@gmail.com

**Keywords:** inhibitors, melanogenesis, tyrosinase

## Abstract

Melanogenesis is a biosynthetic pathway for the formation of the pigment melanin in human skin. A key enzyme, tyrosinase, catalyzes the first and only rate-limiting steps in melanogenesis, and the down-regulation of enzyme activity is the most reported method for the inhibition of melanogenesis. Because of the cosmetically important issue of hyperpigmentation, there is a big demand for melanogenesis inhibitors. This encourages researchers to seek potent melanogenesis inhibitors for cosmetic uses. This article reviews melanogenesis inhibitors that have been recently discovered from natural sources. The reaction mechanisms of the inhibitors on tyrosinase activity are also discussed.

## 1. Introduction to Melanogenesis

**Figure 1:**
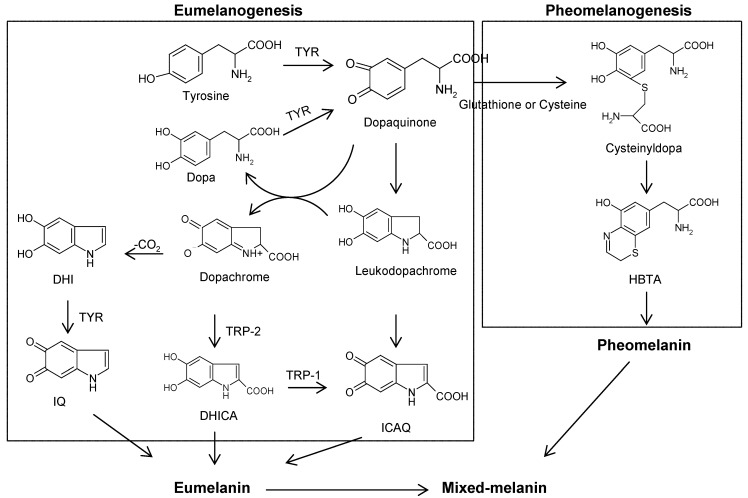
Diagram of melanogenesis [1]. TYR, tyrosinase; TRP; tyrosinase related protein; dopa, 3,4-dihydroxyphenylalanine; DHICA, 5,6-dihydroxyindole-2-carboxylic acid; DHI, 5,6-dihydroxyindole; ICAQ, indole-2-carboxylic acid-5,6-quinone; IQ, indole-5,6-quinone; HBTA, 5-hydroxy-1,4-benzothiazinylalanine.

Human skin color originates from the outermost layer of the skin, the epidermis, where the pigment-producing cells, melanocytes, are localized to produce melanin. The distribution pattern of synthesized melanin by the melanocytes determines the actual color of the skin. Melanin also plays a crucial role in the absorption of the free radicals generated within the cytoplasm and in shielding the host from various types of ionizing radiation, including UV light. Melanin is formed by a process called melanogenesis through a combination of enzymatically catalyzed and chemical reactions. Melanogenesis takes place in special organelles, melanosomes, in the melanocytes. The biosynthetic pathway of melanogenesis has been elucidated, where two types of melanin are synthesized within melanosomes: eumelanin and pheomelanin (Figure 1) [1]. The first step of melanogenesis is initiated with tyrosine oxidation to dopaquinone catalyzed by the key enzyme, tyrosinase. This first step is the only rate-limiting step in melanin synthesis because the remainder of the reaction sequence can proceed spontaneously at a physiological pH value [2]. After dopaquinone formation by tyrosinase, the compound is converted to dopa and dopachrome through auto-oxidation. Dopa is also the substrate of tyrosinase and oxidized to dopaquinone again by the enzyme. Finally, eumelanin is formed through a series of oxidation reactions from dihydroxyindole (DHI) and dihydroxyindole-2-carboxylic acid (DHICA), which are the reaction products from dopachrome. In the presence of cysteine or glutathione, dopaquinone is converted to cysteinyldopa or glutathionyldopa. Subsequently, pheomelanin is formed. Although three enzymes [tyrosinase, tyrosinase-related protein 1 and 2 (TRP1 and TRP2)] are involved in the melanogenesis pathway, only tyrosinase is absolutely necessary for melanogenesis, due to its key role in the process. The enzyme is a glycoprotein located in the membrane of the melanosome with an inner melanosomal domain that contains the catalytic region, followed by a short transmembrane domain and a cytoplasmic domain [3]. The notable feature observed in tyrosinase is that a central copper-binding domain is conserved, which contains strictly conserved amino acid residues, including three histidines [4]. Tyrosinase is produced only by melanocytic cells. Following its synthesis and subsequent processing in the endoplasmic reticulum (ER) and Golgi, it is trafficked to melanosomes, wherein the pigment melanin is synthesized. 

**Figure 2:**
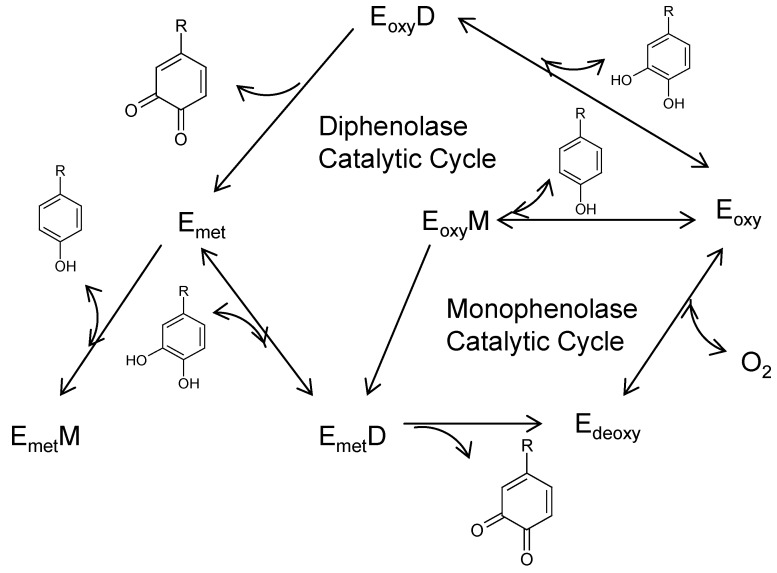
Catalytic cycles of the hydroxylation of monophenol and oxidation of o-diphenol to o-quinone by tyrosinase [1]. E_oxy_, E_met_, and E_deoxy_ are the three types of tyrosinase, respectively. E_oxy_D, E_oxy_M, and E_met_M are E_oxy_-Diphenol, E_oxy_-Monophenol, and E_met_-Monophenol complexes, respectively.

Tyrosinases catalyze the oxidations of both monophenols (monophenolase activity) and o-diphenols (diphenolase activity) into reactive o-quinones (Figure 2). In the formation of melanin pigments, three types of tyrosinase (oxy-, met-, and deoxytyrosinase) with different binuclear copper structures of the active site are involved. In the monophenolase cycle, the monophenol can react only with the oxy form and be catalyzed to a coordinated *o*-diphenol, which is oxidized to the o-quinone, resulting in a deoxy form ready for further dioxygen binding. Oxytyrosinase is, then, regenerated after the binding of molecular oxygen to deoxytyrosinase. If only o-diphenol is present (the diphenolase cycle), both the oxy and met forms react with o-diphenol, oxidizing it to the o-quinone. O-diphenol binds to the oxy form and is oxidized to o-quinone, yielding the met form of the enzyme. The latter form transforms another o-diphenol molecule into o-quinone and is reduced to the bicuprous deoxy form.

## 2. Regulation of Melanogenesis

**Figure 3:**
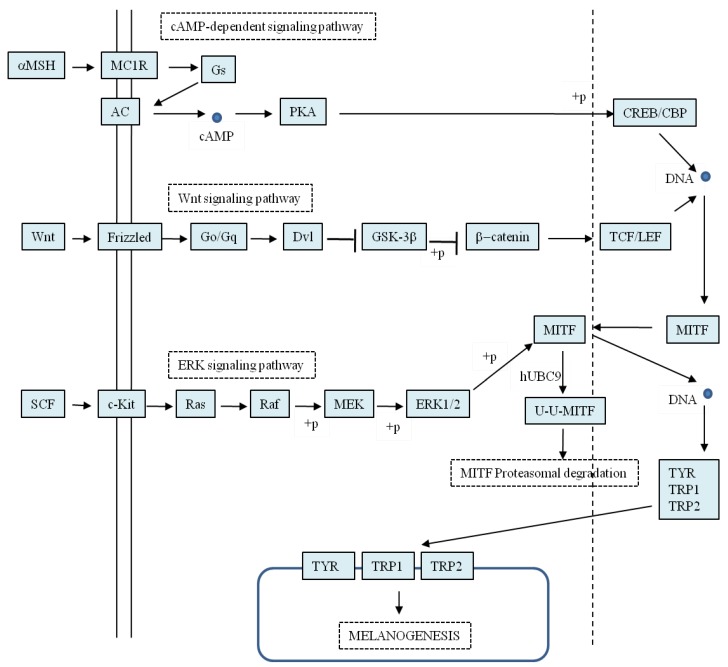
Three common signal pathways involved in the regulation of melanogenesis. (Modified from melanogenesis pathway map of Kyoto Encyclopedia of Genes and Genomes) [9].

Regulation of melanogenesis in mammals is controlled at different levels and is complex at each level [5,6]. During embryo development, melanocytes are initially derived from the neural crest and migrate throughout the embryo to the target sites. The migration patterns are under stringent genetic control, and this is the first level of melanogenesis regulation. Melanogenesis is also regulated at the cellular level *via* the controlling formation of melanosomes, which can be produced in varying sizes, numbers, and densities depending on melanin content. Finally, melanogenesis is regulated at the subcellular level where the gene expression encoded by the melanogenesis-related enzymes, including tyrosinase, TRP1 and TRP2, is regulated by intracellular pathways. These signal pathways are initiated by a variety of hormones, including interleukins, interferons, growth factors, and prostaglandins, which determine not only the quantity but also the quality of the synthesized melanin. The hormones provide the complex signals that respond to UV exposure or other environmental stimulations. Figure 3 shows the three most commonly known signal pathways involved in the regulation of melanogenesis. All three signal pathways involve microphthalmia-associated transcription factor (MITF), which is a transcription factor with the structural domain of basic helix-loop-helix leucine zipper. In addition to being involved in the survival, proliferation, and differentiation of melanocytes, MITF is the master regulator of melanogenesis in melanocytes *via* binding to the M box of a promoter region and regulating the gene expression of tyrosinase, TRP-1, and TRP-2 [7,8]. The up-regulation of MITF activity activates the expression of the melanogenesis-related enzymes, thus stimulating melanogenesis. In contrast, the down-regulation of MITF activity depresses the expression of the related enzymes, thereby inhibiting melanogenesis.

Alpha melanocyte-stimulating hormone (α-MSH), a peptide derived from proopiomelanocortin (POMC), regulates melanogenesis *via* a cyclic adenosine monophosphate (cAMP)-dependent pathway [10,11]. When binding to its receptor, melanocortin receptor 1 (MC1R), on the membrane of melanocytes, the hormone activates adenylate cyclase (AC) to produce cAMP as an intracellular second message *via* a G-protein-coupled receptor (GPCR)-type activation. cAMP activates protein kinase A (PKA), which then activates the gene expression of MITF *via* phosphorylation of the cAMP response element-binding protein (CREB). Finally, MITF efficiently activates the melanogenesis-related enzymes and stimulates melanogenesis. Once α-MSH binds to MC1R, up to a 100-fold increase in melanogenesis attends. In addition to α-MSH, other POMC-derived peptides, such as β-MSH and adrenocorticotropic hormone (ACTH), also stimulate melanogenesis *via* the same pathway. 

Another signal pathway also targeting the gene expression of MITF is the Wnt signal pathway. A key control in this pathway is the level of intracellular β-catenin. In the absence of a Wnt signal, β-catenin is sequentially phosphorylated by glycogen synthase kinase-3β (GSK-3β), and the phosphorylated β-catenin is recognized by an ubiquitin ligase complex, resulting in the degradation of β-catenin *via* an ubiquitin-dependent mechanism. In contrast, activation of the Wnt pathway negatively regulates GSK-3β, leading to the accumulation of cytoplasmic β-catenin, which translocates to nuclei and forms a complex with both T-cell factor (TCF) and lymphocyte enhancer factor-1 (LEF) to up-regulate expression of the MITF gene [12,13,14]. Hence, activation of the Wnt pathway stimulates melanogenesis *via* the up-regulation of MITF activity.

In contrast to the regulation of gene expression of MITF by α-MSH and Wnt signal pathways, the extracellular signal-regulated kinase (ERK) pathway regulates melanogenesis *via* the degradation of the MITF protein [15]. Previous studies have shown that ERK activation phosphorylates MITF at serine 73, which is followed by MITF ubiquitination and proteasome-mediated degradation [16,17]. Therefore, activation of the ERK pathway would inhibit melanogenesis due to the down-regulation of the MITF activity. In addition, some reports have emphasized the important roles of c-Kit in the ERK pathway [18,19]. 

## 3. Natural Melanogenesis Inhibitors Acting Through the Down-Regulation of Tyrosinase Activity

Although melanin has mainly a photoprotective function in human skin, excess melanin production or abnormal distribution can cause irregular hyperpigmentation of the skin. Exposure to certain drugs and chemicals as well as the existence of certain disease states, such as melasma and age spots, can result in hyperpigmentation. Moreover, recent investigation suggests that many melonogenesis disorders are reported to have links with neurodegenerative diseases, including Parkinson’s and Alzheimer’s. Therefore, there is a big demand for melanogenesis inhibitors in order to develop therapies or prevent hyperpigmentary disorders [20]. This provides the impetus for researchers to find new and potent melanogenesis inhibitors. 

For the screening of antimelanogenic effects, B16F10 murine melanoma cells were widely used, probably because they are relatively easy to culture *in vitro*, and they share most of the melanogenic mechanisms of normal human melanocytes. In addition, arbutin, which is a hydroquinone derivative widely used in cosmetics as a hypopigmenting agent, is usually used as a positive control. As a result, many new melanogenesis inhibitors have been discovered in recent years. Several review articles have discussed in-depth some well-known and commercial skin-whitening agents, such as hydroquinone, kojic acid, arbutin, magnesium ascorbyl phosphate, licorice extract, aloesin, azelaic acid, soybean extract, and niacinamide [21,22,23,24,25,26,27]. The present review surveys melanogenesis inhibitors discovered after 2008. Because the down-regulation of tyrosinase activity is the most reported mechanism for melanogenesis inhibition, melanogenesis inhibitors, whose reaction mechanisms are unknown or do not target tyrosinase activity, are excluded in the present review. In addition, crude extracts containing unknown ingredients from various plants, which give little information about the detailed reaction mechanisms, are also not discussed here. On the other hand, melanogenesis inhibitors from natural sources usually attract more attention compared to chemically synthesized compounds due to consumer cosmetic demand. Therefore, the present review focuses mainly on newly isolated compounds from natural sources that display melanogenesis inhibitory activity through the down-regulation of tyrosinase activity.

### 3.1. Directly Inhibiting Tyrosinase Catalytic Activity

The most common target for melanogenesis inhibitors is direct inhibition of tyrosinase catalytic activity. Moreover, in the view of clinical applications in cosmetics and pharmaceuticals, tyrosinase inhibitors are the most popular and wildly-used hypopigmenting agents. Because tyrosinase is produced only by melanocytic cells, tyrosinase inhibitors have highly specific targeting to melanogenesis in the cells without other side effects. In contrast, those melanogenesis inhibitors targeting to the tyrosinase gene expressions or protein degradations are rarely used as clinical hypopigmenting agents, due to their non-specific and global effects *via* intracellular signaling pathways. Hence, searching for new melanogenesis inhibitors based on direct inhibition of tyrosinase catalytic activity seems to still be the major field of interest for further study.

**Figure 4:**
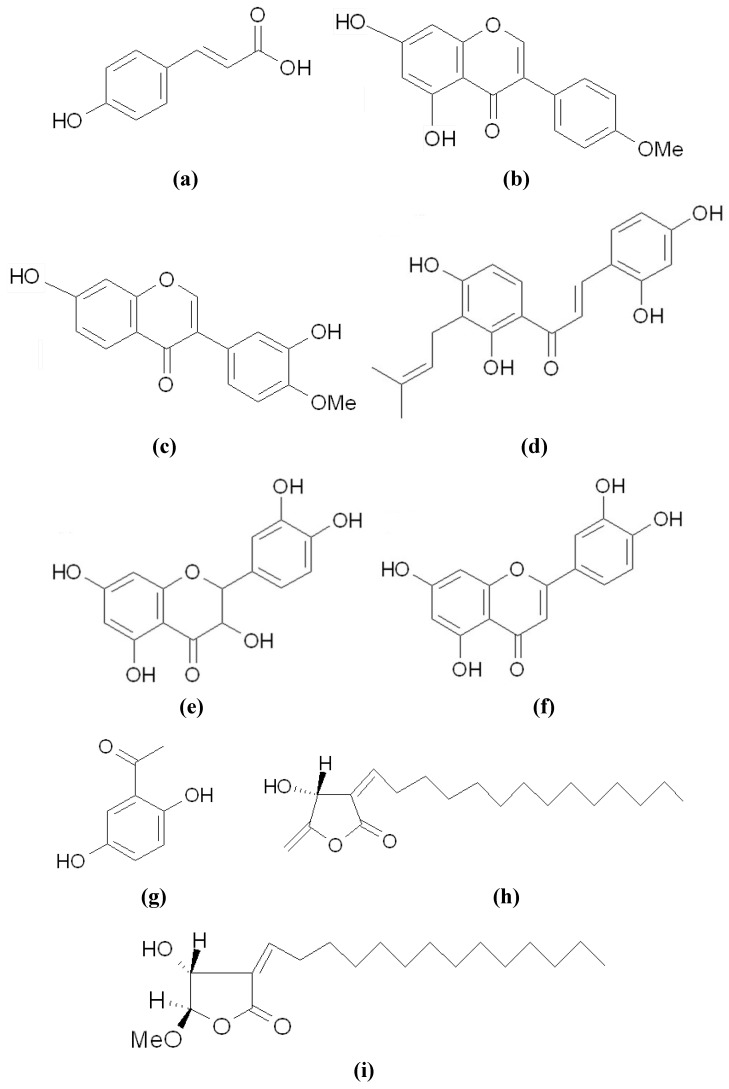
Chemical structures of some tyrosinase inhibitors. (**a**) p-Coumaric Acid; (**b**) Biochanin A; (**c**) Calycosin; (**d**) TMBC; (**e**) Taxifolin; (**f**) Luteolin; (**g**) 2,5-DHAP; (**h**) Linderanolide B; (**i**) Subamolide A.

Numerous reviews have described the inhibition of tyrosinase activity, many of them using mushroom tyrosinase as a model [1,28,29,30]. The commercial availability of mushroom tyrosinase plays a critical role in tyrosinase inhibitor studies, and most research has been conducted with this enzyme, which is well-studied and easily purified from the mushroom *Agaricus bisporus*. However, the use of mushroom tyrosinase for this purpose can be problematic because it differs significantly from mammalian tyrosinases in several respects. The mushroom tyrosinase is a cytosol enzyme, while the human tyrosinase is membrane bonded [30]. In addition, mushroom tyrosinase is a tetramer in contrast to the monomer type of the human enzyme, which is highly glycosylated during its complex maturation process [31].

p-Coumaric Acid (p-CA) (Figure 4a) is a classic example of a tyrosinase inhibitor, which shows highly different inhibitions of the activities of mushroom tyrosinase and mammalian tyrosinase. The compound is a common secondary metabolite of plants and has been proven to inhibit melanogenesis in α-MSH-stimulated B16 cells [32]. Due to its very close chemical structure to tyrosine, p-CA may compete with tyrosine for active sites on the enzyme. The inhibitory effect of p-CA on melanogenesis is stronger than structurally similar compounds, such as cinnamic acid and caffeic acid, implying that p-CA has an optimal structure for the inhibition of mammalian tyrosinase [33]. However, despite the fact that p-CA performs excellent inhibition on human or murine tyrosinase, p-CA is a much weaker inhibitor of mushroom tyrosinase [34]. 

Another example showing the limitation of applying information regarding the discovered inhibitors of mushroom tyrosinase on mammalian melanogenesis is the common natural flavonoid, quercetin, which has been proven to be a potent mushroom tyrosinase inhibitor [35]. However, quercetin was reported to enhance melanogenesis in mouse melanoma cells, human melanoma, and normal melanocytes by increasing the activity and synthesis of tyrosinase [36,37]. From the two examples described above, the inhibition of mushroom tyrosinase activity does not correlate well with the inhibition of cellular tyrosinase or of melanin production in melanocytes. In fact, Song *et al*. have demonstrated that the inhibition of mushroom tyrosinase activity does not represent a useful marker for cellular melanin formation [38]. Hence, the studies of tyrosinase inhibitors using mushroom tyrosinase only have been omitted from this review.

Among the newly discovered tyrosinase inhibitors, flavonoids occupy most portions. We recently identified biochanin A (4'-methoxy-5,7-dihydroxyisoflavone) (Figure 4b) from *Trifolium pretense* as a new melanogenesis inhibitor, which acts by directly inhibiting tyrosinase activity without affecting gene expression [39]. Another isoflavone, calycosin (4'-methoxy-3',7-dihydroxyisoflavone) (Figure 4c), has a structure similar to biochanin A, and has also been reported to show both anti-melanogenic and anti-tyrosinase activities in mouse melanoma cells [40]. However, in addition to tyrosinase inhibitory activity, calycosin also reduces tyrosinase gene expression. The results reveal that the numbers and positions of the functional groups on the skeleton of isoflavones strongly affect their bioactivity. 2,4,2',4'-Tetrahydroxy-3-(3-methyl-2-butenyl)-chalcone (TMBC) (Figure 4d), which was isolated from the stem of *Morus nigra*-known as black mulberry, inhibited melanogenesis by direct inhibition of cellular tyrosinase activity [41]. It is quite interesting to find that tyrosinase gene expression in the TMBC-treated cells was up-regulated in a dose-dependent manner, although melanogenesis in the cells was inhibited. It has been reported that treatment with a potent tyrosinase inhibitor can lead to the up-regulation of the tyrosinase mRNA level as a compensatory mechanism to maintain melanin homeostasis [42]. Similar situations have also occurred in melanogenesis inhibitions with treatments of taxifolin (Figure 4e) and luteolin (Figure 4f) [43]. The two compounds exhibited potent melanogenesis inhibition *via* inhibitory activity on cellular tyrosinase activity, despite increasing tyrosinase protein levels in the cells. 

In order to examine the effectiveness of the newly discovered inhibitors as potential human therapeutic drugs, evaluating anti-melanogenesis activity *in vivo* is essential. Zebrafish have recently been established as a new *in vivo* model for the evaluation of hypopigmentation activity of melanogenic regulatory compounds [44]. This animal model system has advantages, including easy maintenance and handling, and high efficiency in drug penetration through the skin. For these reasons, the zebrafish model was used as an *in vivo* system to evaluate the inhibition of melanogenesis by some newly found inhibitors, including 2,5-dihydroxyacetophenone (2,5-DHAP) (Figure 4g) isolated from *Cynanchum bungei* [45], linderanolide B (Figure 4h), and subamolide A (Figure 4i) isolated from *Cinnamomum subavenium* [46]. All three compounds exhibited potent hypopigmenting activity in the zebrafish model, while 2,5-DHAP also showed skin-whitening activity in a mouse model. 

### 3.2. Accelerating Tyrosinase Degradation

In eukaryotic cells, selective elimination of proteins is an important mechanism by which physiological processes are regulated. The synthesis and the degradation of tyrosinase are tightly coupled to its function, and are influential parameters that regulate melanin synthesis. Tyrosinase is initially synthesized and processed in the ER, following which it is transferred to the Golgi apparatus, where it is matured by complex sugar modification [31]. After synthesis, the protein exists for a while before it is degraded. Until now, two tyrosinase degradation pathways, proteasomal and lysosomal, have been reported to be involved in the turnover of tyrosinase protein [47,48]. In instances where a substance accelerates the degradation of tyrosinase protein by one of the two pathways, the compound exhibits anti-melanogenesis activity.

**Figure 5:**
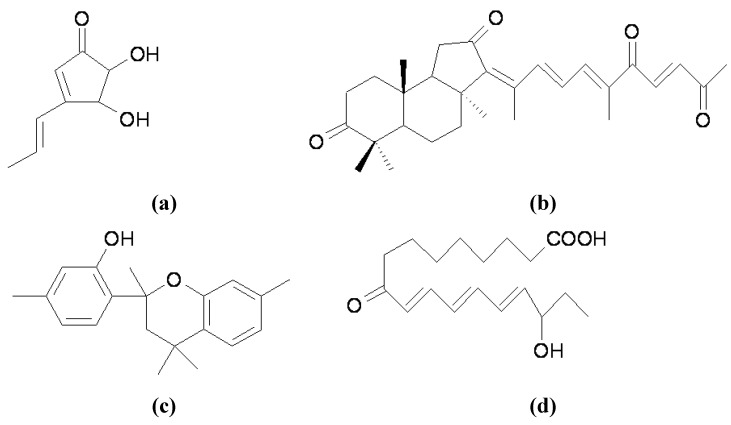
Melanogenesis inhibitors with activity on accelerating tyrosinase degradation. (**a**) Terrein; (**b**) Geoditin A; (**c**) Inulavosin; (**d**) CFAB.

Many short-lived proteins related to cell cycle progression, signal transduction, transcriptional regulation, receptor downregulation, and endocytosis are selectively removed by the ubiquitin-mediated proteasomal degradation system, which is a multicatalytic proteinase complex that selectively degrades intracellular ubiquitylated proteins [49]. According to the review by Ando *et al*. [6], several melanogenesis inhibitors, such as linoleic acid [50], 25-hydroxycholesterol [51], phospholipase D2 [52], and phenylthiourea [53], negatively regulate tyrosinase activity by accelerating its ubiquitin-dependent degradation. Consequently, these reagents exhibit inhibitory effects on cell pigmentation. In addition, Park *et al*. recently reported a new melanogenesis inhibitor, terrein (Figure 5a), a novel fungal metabolite, which was originally isolated from the *Penicillium* species [54]. Terrein was first found to strongly decrease tyrosinase production by down-regulating MITF *via* ERK activation, and thus inhibiting melanin synthesis [55]. However, the authors recently found that terrein also induced long-term suppressive effects against the synthesis of melanin through ubiquitin-dependent proteasomal degradation [56]. This dual and long-lasting effect of terrein on tyrosinase contributes to its strong hypopigmentary effect.

On the other hand, tyrosinase has common targeting motifs for melanosomes and lysosomes, making it possible to be mistargeted to lysosomes for degradation [48]. Recently, geoditin A (Figure 5b), which is an isomalabaricane triterpene isolated from the South China Sea Sponge, *Geodia japonica*, was reported to exhibit melanogenesis inhibition *via* a post-translational modification in the ER and the degradation of tyrosinase in the lysosome [57,58]. Inulavosin (Figure 5c), a melanogenesis inhibitor isolated from *Inula nervosa*, also reduced melanogenesis by accelerating tyrosinase degradation without affecting the enzymatic activities, transcription of tyrosinase, or TRP1 in B16 melanoma cells [59]. Moreover, the study showed that the degradation of tyrosinase induced by inulavosin is associated with lysosomes, but not proteasomes.

In addition to the two tyrosinase degradation pathways described above, Fujita *et al*. recently isolated 16-hydroxy-9-oxo-10E,12E,14E-octadecatrienoic acid, also known as Corchorifatty acid B (CFAB) (Figure 5d), from the ethanol extracts of the aerial parts of *Melissa officinalis*, and found that the compound specifically accelerates the rate of tyrosinase decrease without any effect on tyrosinase activity or transcription [60]. However, unexpected results suggested that the CFAB-induced decrease in tyrosinase did not occur in the lysosomes or proteasomes; neither were proteasome inhibitors nor lysosomal inhibitors able to inhibit a CFAB-induced decrease in tyrosinase. To answer the question “Where did tyrosinase go?” the authors proposed an attractive model, exosomal secretion, for the clearance of tyrosinase by CFAB. However, further careful analysis is required in order to understand fully the mechanism of CFAB-induced tyrosinase decrease.

### 3.3. Inhibiting Tyrosinase Gene Expression via Mitf

**Figure 6:**
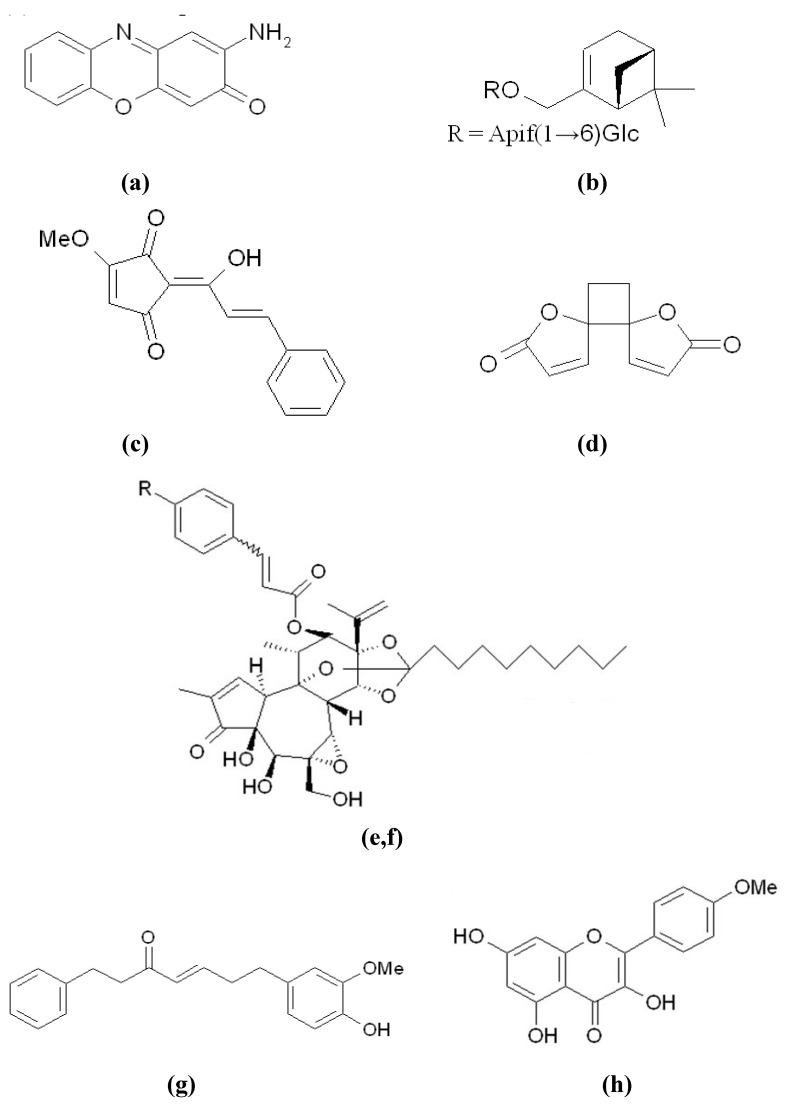

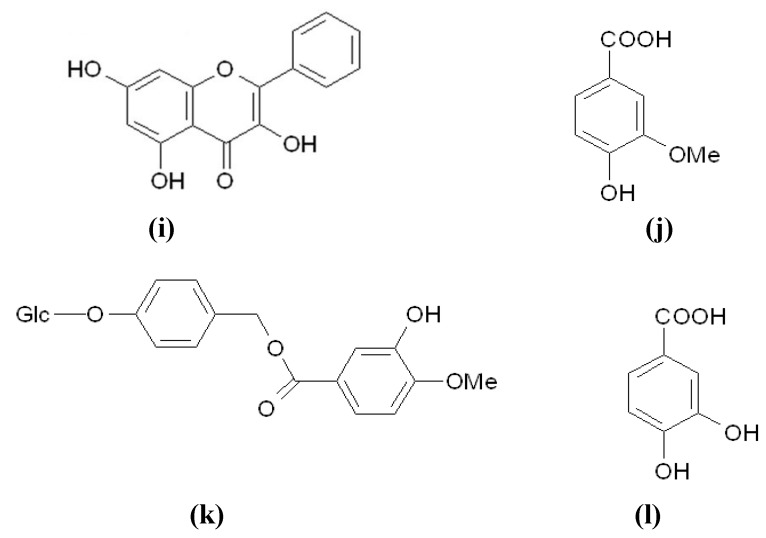
Melanogenesis inhibitors with activity on the down-regulation of MITF protein. (**a**) 2-Amino-3*H*-phenoxazin-3-one; (**b**) Myrtenol 10-O-[β-D-apiofuranosyl-(1→6)-β-D-glucopyranoside]; (**c**) Lucidone; (**d**) Anemonin; (**e**, **f**) Hirsein A (R = H) and Hirsein B (R = OH); (**g**) 7-(4"-Hydroxy-3"-methoxyphenyel)-1-phenylhept-4-en-3-one; (**h**) Kaempferide; (**i**) Galangin; (**j**) Vanillic acid; (**k**) Origanoside; (**l**) Protocatechuic acid.

As described in Section 2 of this paper, MITF is the master regulator of melanogenesis in melanocytes *via* binding to the M box of a promoter region and regulating the gene expression of tyrosinase, TRP-1, and TRP-2. The down-regulation of MITF activity depresses the expression of the related enzymes, thereby inhibiting melanogenesis. In recent years, many melanogenesis inhibitors, acting through the down-regulation of MITF activity, have been discovered. The mechanism linked to one of the three MITF-related pathways of melanogenesis regulation, shown in Figure 3, has been studied in detail. These inhibitors will be discussed in the following subsections. On the other hand, some studies have shown only the results of the down-regulation of the MITF protein by either a western blotting method or a reverse-transcription polymerase chain reaction (RT-PCR) method, and have not produced a clear explanation of MITF inhibition. Hence, we cannot explain the detailed pathway involved in MITF repression by the inhibitors. These inhibitors include 2-amino-3*H*-phenoxazin-3-one (Figure 6a) from the mushroom *Agaricus bisporus* [61], myrtenol 10-O-[β-d-apiofuranosyl-(1→6)-β-d-glucopyranoside] (Figure 6b) from the leaves of *Momordica charantia* [62], lucidone (Figure 6c) from the fruits of *Lindera erythrocarpa* [63], anemonin (Figure 6d) from the leaves of *Clematis crassifolia* [64], hirsein A (Figure 6e) and hirsein B (Figure 6f) from the leaves of *Thymelaea hirsuta* [65,66], 7-(4''-hydroxy-3''-methoxyphenyl)-1-phenylhept-4-en-3-one (Figure 6g), kaempferide (Figure 6h), and galangin (Figure 6i) from the rhizomes of *Alpinia officinarum* [67], and vanillic acid (Figure 6j) [68], origanoside (Figure 6k) [69], and protocatechuic acid (Figure 6l) [70] from *Origanum vulgare*. Despite there being no detailed explanation for the mechanism regarding MITF inhibition by the inhibitors, vanillic acid and origanoside both show excellent hypopigmentation activity in *in vivo* mice systems and are good candidates in skin-whitening cosmetics applications. 

#### 3.3.1. Acting Through the cAMP-Dependent Pathway

**Figure 7:**
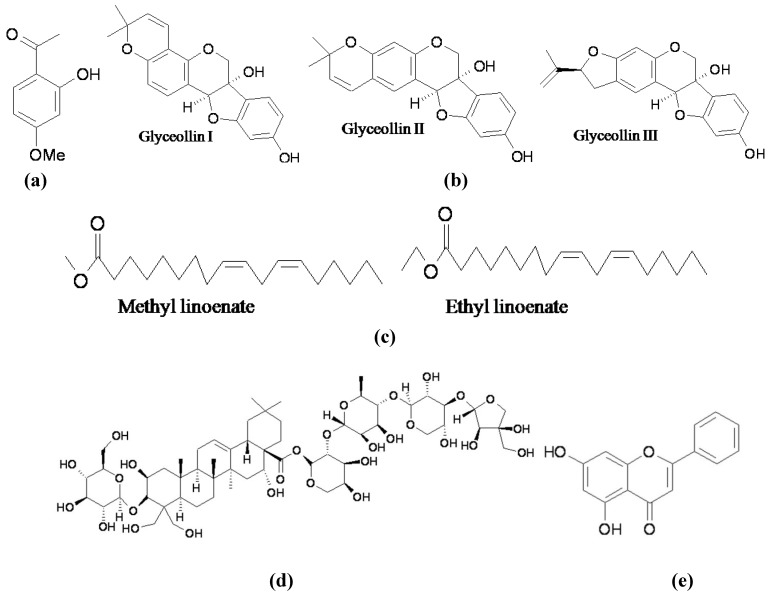
Melanogenesis inhibitors acting through the cAMP-dependent pathway. (**a**) Paeonol; (**b**) Glyceollin; (**c**) Methyl/ethyl linoenate; (**d**) Platycodin D; (**e**) Chrysin.

The major signaling pathway leading to melanin synthesis appears to be the stimulation of AC activity followed by an increase in the cAMP level. cAMP increases the expression of MITF through activation of the PKA and CREB, which in turn stimulates tyrosinase gene expression to allow melanin synthesis (cAMP-dependent pathway in Figure 3). Therefore, agents trying to block the signal pathway would exhibit melanogenesis inhibitory activity. Paeonol (Figure 7a), a major component of Moutan Cortex, has been reported to effectively reduce melanogenesis [71]. In an advanced study, Bu *et al*. found that the compound markedly reduced mRNA, and the protein expression of tyrosinase and MITF in B16 melanoma cells. Furthermore, paeonol treatment inhibited the phosphorylation of CREB. These results suggest that paeonol inhibits phosphorylated CREB, leading to the inactivation of MITF and tyrosinase expression, and a consequent reduction in melanin synthesis [72]. Similarly, glyceollins (Figure 7b), one of a group of phytoalexins produced in soybeans under stress conditions, inhibited melanin production in B16 melanoma cells by inhibiting intracellular cAMP levels and reducing MITF expression, followed by a subsequent decrease in both mRNA and protein levels of tyrosinase [73]. In addition, Huh *et al*. found that the methyl/ethyl linoenate (Figure 7c) from *Oxalis triangularis* exhibited melanogenesis inhibition, which was also mediated by a reduction in the cAMP level in cells [74].

In the last year, platycodin D (Figure 7d), a triterpene saponin isolated from Platycodi Radix, the root of *Platycodon grandiflorum*, was found to specifically inhibit melanin synthesis through the down-regulation of cAMP, phosphorylation of CREB, and expression of MITF and its associated genes [75]. On the other hand, a molecular docking study to elucidate the binding site structure for 5,7-dihydroxyflavone (chrysin, Figure 7e) from propolis on the AC enzyme structure implies the specific binding of chrysin to AC. The authors demonstrated further that chrysin directly inhibits AC activity, down-regulates the cAMP-production pathway, and consequently inhibits melanogenesis [76]. 

#### 3.3.2. Acting through the Wnt Pathway

**Figure 8:**
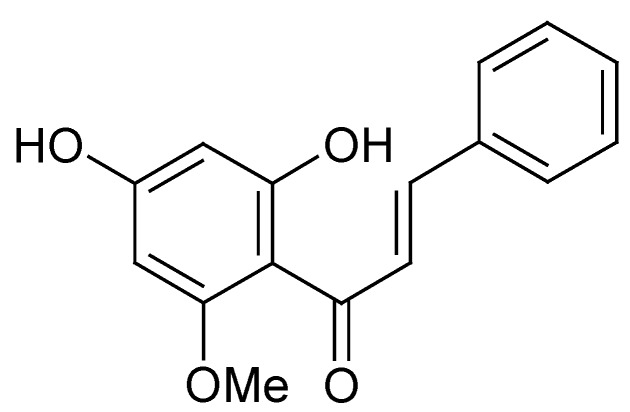
Chemical structure of cardamonin.

Although it was proposed in 2000 that the Wnt pathway is involved in the regulation of melanogenesis (Figure 3), few melanogenesis inhibitors were found to react through this pathway. Recently, Cho *et al*. reported that cardamonin (Figure 8), a calchone from *Aplinia katsumadai*, promoted the degradation of intracellular β-catenin while suppressing melanogenesis by down-regulating the expression of MITF and tyrosinase [77]. In fact, cardamonin is the only natural melanogenesis inhibitor discovered to act through the Wnt pathway in recent years, despite some chemically synthetic imidazole derivatives also being proven to inhibit melanogenesis *via* the same pathway [13]. Therefore, further research to find new melanogenesis inhibitors that belong to the Wnt-type is warranted. 

#### 3.3.3. Acting through the ERK Pathway

**Figure 9:**
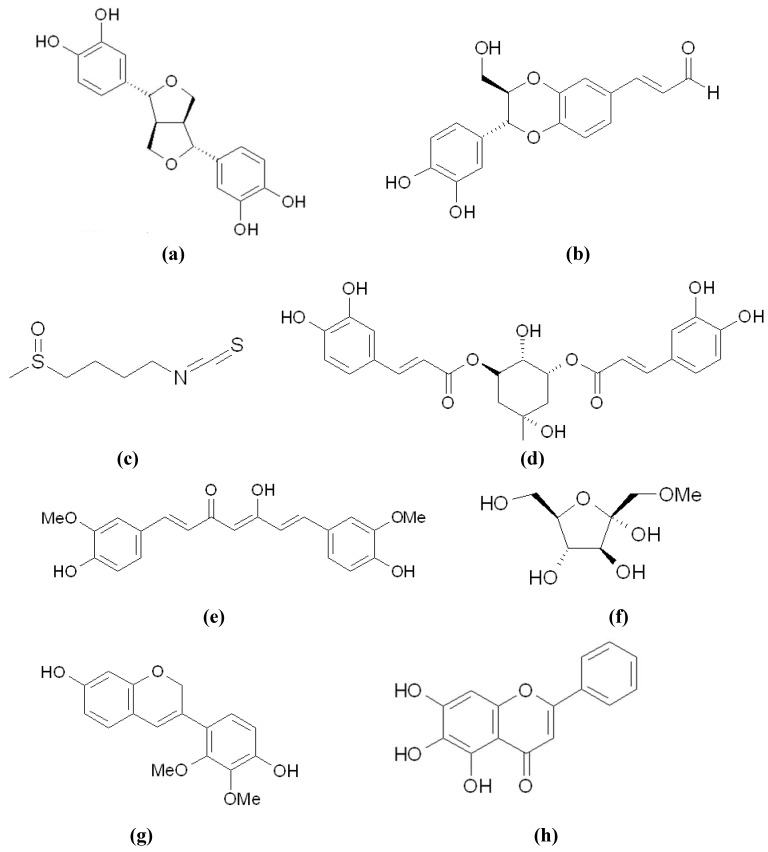
Melanogenesis inhibitors acting through the ERK pathway. (**a**) 3,3-Bisdemethylpinoresinol; (**b**) Americanin A; (**c**) Sulporaphane; (**d**) MDQ; (**e**) Curcumin; (**f**) O-Methyl-fructofuranose; (**g**) Haginin A; (**h**) Baicalein.

A total of eight melanogenesis inhibitors (Figure 9) are included and discussed in the present subsection. All of the inhibitors have been demonstrated to exert anti-melanogenesis activity through the activation of the ERK pathway, then MITF ubiquitination and degradation, followed by down-regulation of tyrosinase activity (ERK pathway in Figure 3). Although similar mechanisms regarding the activation of the ERK pathway by inhibitors exist, there are two different points in the inhibitory reactions among them. 

First, the status related to the activity of a p38 protein is different for some inhibitors. In addition to the ERK, another member of the MAP kinase family, p38 MAPK, is thought to play an important role in the regulation of melanogenesis. However, there are different explanations for the reaction mechanism of p38 on the regulation of melanogenesis. Ye *et al*. reported that p38 phosphorylates and activates MITF, thus stimulating melanin production [78]. Therefore, inhibiting p38 phosphorylation would reduce MITF activity and thus melanin biosynthesis. The hypothesis is supported by studies of melanogenesis inhibitors, including 3,3′-bisdemethylpinoresinol (Figure 9a) and americanin A (Figure 9b) from *Morinda citrifolia* [79], sulporaphane (Figure 9c) from broccoli [80], and methyl 3,5-dicaffeoyl quinate (MDQ) (Figure 9d) from the leaves of *Kalopanax pictus* [81]. The four inhibitors decrease melanogenesis through the suppression of tyrosinase expression resulting from the down-regulation of MITF activity. With respect to the reasons for the MITF down-regulation, besides activating ERK, the inhibitors were all found to inhibit p38 phosphorylations. On the other hand, a recent study showed that p38 promotes the degradation of melanogenesis-related enzymes, thus inhibiting melanin production [82]. In this scenario, activating p38 phosphorylation would reduce melanogenesis-related enzymes and thus inhibit melanin biosynthesis. The findings of curcumin (Figure 9e), which is a polyphenol from the rhizome of *Curcuma longa* and inhibits melanogenesis in both mouse B16 cells [83] and human melanocytes [84] through activation of both ERK and p38 MAPK signaling pathways, are consistent with the second hypothesis. Hence, the detailed reaction mechanism of p38 on melanogenesis regulations is not fully understood and needs to be studied in the future.

Second, the conditions regarding Akt/PKB activation are different for some inhibitors. Recently, it has been suggested that AKt is involved in the negative regulation of melanogenesis, and that the activation of Akt results in the inhibition of melanogenesis [85,86]. In addition to the activations of both ERK and p38 proteins, the curcumin described above was found to activate Akt when presenting anti-melanogenesis activity in both mouse B16 cells and human melanocytes [83,84]. In addition, *O*-methyl-fructofuranose (Figure 9f) from the *Schisandra chinensis* fruit [87] and haginin A (Figure 9g) from *Lespedeza cyrtobotrya* [88] were also found to activate phosphorylations of both ERK and Akt in performing melanogenesis inhibition. All of these reports reflect the activation of Akt as being involved in the inhibition of melanogenesis. However, the melanogenesis inhibitors, MDQ (Figure 9d) [81] and baicalein (Figure 9h) [89], were found not to activate Akt, although they inhibited melanogenesis through MITF degradation mediated by ERK activation. Hence, the roles and importance of Akt in regulating melanogenesis has yet to be determined. 

### 3.4. Other Inhibitors Acting through the Down-Regulation of Tyrosinase Protein

**Figure 10:**
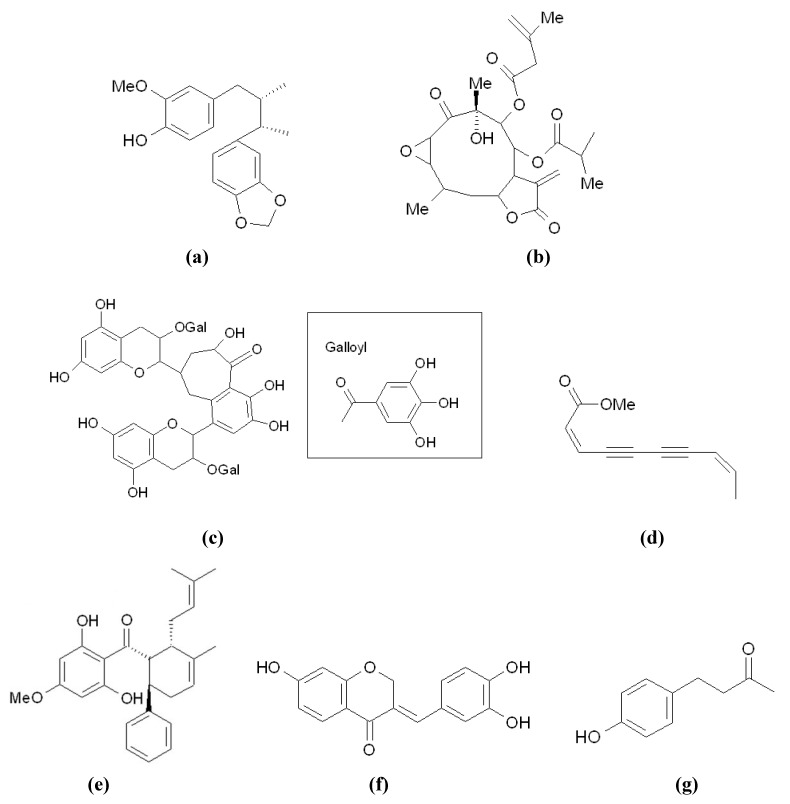

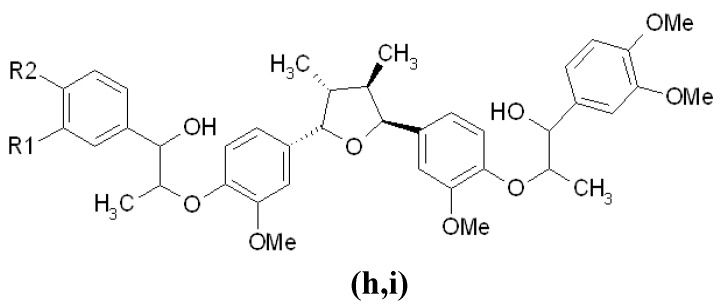
Melanogenesis inhibitors acting on the down-regulation of tyrosinase protein. (**a**) Macelignan; (**b**) 2,3-Epoxyjuanislamin; (**c**) Theaflavin-3,3'-digallate; (**d**) (2Z, 8Z)-Matricaria acid methyl ester; (**e**) Panduratin A; (**f**) Sappanone A; (**g**) Raspberry ketone; (**h**,**i**) Manassantin A (R_1_=R_2_=OMe) and Manassantin B (R_1_-OCH_2_O-R_2_).

In this final part of Section 3, some melanogenesis inhibitors recently discovered from natural sources are discussed. All of the inhibitors were demonstrated to decrease melanogenesis *via* the down-regulation of both tyrosinase protein and its activity in the treated cells. Through evaluation by a RT-PCR method, some of them were also shown to inhibit tyrosinase gene expression. However, there is no further evidence regarding the reaction mechanism in these studies, and the inhibitors therefore cannot be classified into the sections described above. They include macelignan (Figure 10a) from *Myristica fragrans* [90], 2,3-epoxyjuanislamin (Figure 10b) from the leaves of *Calea urticifolia* [91], a black tea component theaflavin-3,3'-digallate (Figure 10c) [92], (2*Z*,8*Z*)-matricaria acid methyl ester (Figure 10d) from *Erigeron breviscapus* [93], panduratin A (Figure 10e) from *Kaempferia pandurata* [94], sappanone A (Figure 10f) from *Caesalpinia sappan* [95], raspberry ketone (Figure 10g) from *Rheum officinale* [96], and manassantin A (Figure 10h) and B (Figure 10i) from *Saururus chinensis* [97]. 

Although potent *in vitro* inhibitory activity of the above inhibitors has been proven, rare inhibitors were determined for the hypopigmenting activity *in vivo*, with the exception of raspberry ketone, the hypopigmenting activity of which was proven *via* both zebrafish and mice *in vivo* systems. In fact, raspberry ketone has been widely used for a long time in both perfumery and cosmetics, to impart a fruity aroma. It is generally used in jasmine, tuberose, and gardenia fragrance formulas. Moreover, the Federal Emergency Management Agency (FEMA) gave raspberry ketone a “GRAS” (generally regarded as safe) status in 1965 [96]. Hence, the first finding of the compound to inhibit melanogenesis will expand its applications in cosmetics. On the other hand, it is worth noting that sappanone A is the only homoisoflavanone to be identified with melanogenesis inhibitory activity until now. In view of its structure and activity relationship (SAR), it can be surmised that other homoisoflavanones also act in a similar manner, but this has not yet been evaluated. Therefore, it seems worthwhile to search for new melanogenesis inhibitors in other homoisoflavanones-containing plants.

**Figure 11:**
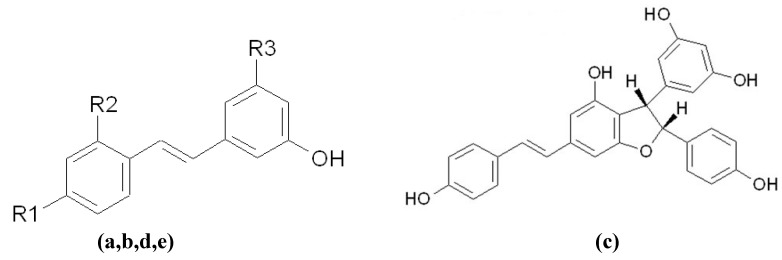
Chemical structures of resveratrol derivatives. (**a**,**b**,**d**,**e**) Resveratrol/Oxyresveratrol/ Piceid/Mulberroside A. (**a**) Resveratrol: R_1_=R_3_=OH, R_2_=H; (**b**) Oxyresveratrol: R_1_=R_2_=R_3_=OH; (**d**) Piceid: R_1_=OH, R_2_=H, R_3_=OGlc; (**e**) Mulberroside A: R_1_=R_3_=OGlc, R_2_=OH. (**c**) Gnetin C.

Before concluding the present section, a group of melanogenesis inhibitors related to the derivatives of resveratrol warrant description. Resveratrol (Figure 11a) is widely distributed in nature in, for example, in the skin of grapes. Both resveratrol and its analog, oxyresveratrol (Figure 11b), were initially recognized as potent tyrosinase inhibitors [98], while the inhibitory mechanism of resveratrol was recently demonstrated to be a K_cat_ (suicide substrate) type [99]. However, Newton *et al*. reported that melanogenesis inhibition by resveratrol on human melanocyte takes place by accelerating the degradation of tyrosinase [48]. Therefore, at least two mechanisms underpin the inhibition of tyrosinase activity by resveratrol. Another interesting melanogenesis inhibitor is gnetin C (Figure 11c), which is a resveratrol dimer from *Gnetum gnemon*. Gnetin C inhibits melanogenesis by directly inhibiting tyrosinase activity and antioxidative properties [100]. In addition, two glycosides of hydroxylstilbene, including a glycoside of resveratrol, piceid (Figure 11d), from *Polygonum cuspidatum* [101] and a glycoside of oxyresveratrol, mulberroside A (Figure 11e), from *Morus alba* [102], were proven to inhibit melanogenesis through the down-regulation of the MITF protein. It must be said that, from the author’s perspective, it is amazing to locate the multiple inhibitory mechanisms linked to resveratrol-related melanogenesis inhibitors.

## 4. Conclusions 

Over the last few years, knowledge of melanocyte biology and the processes underlying melanin synthesis have made remarkable progress, opening paths in the identification of new melanogenesis inhibitors. In addition to the direct inhibition of tyrosinase catalytic activity, other approaches to melanogenesis inhibition include the acceleration of tyrosinase degradation and the inhibition of tyrosinase mRNA transcription *via* a reduction of MITF activity, resulting from decreasing cellular cAMP levels, the accumulation of cytoplasmic β-catenin, or activation of ERK. Accordingly, a huge number of inhibitors acting *via* those alternative approaches have been successfully identified. 

On the other hand, in terms of industrial applications, the use of inhibitors is fundamental to the cosmetic industry due to their skin-whitening effect. However, most inhibitors described in the present review have rarely been incorporated in topically applied cosmetics or cosmeceuticals, often due to a lack of evaluation of hypopigmentation activity in *in vivo* systems or parallel human clinical trials. Since a huge number of melanogenesis inhibitors have been developed, the need to clarify the viability of these inhibitors in terms of their skin-whitening efficiency has become more urgent. In conclusion, more concrete studies of the identified inhibitors from a human clinical point of view are required.
